# Real-time satellite monitoring of the 2024–2025 dyke intrusion sequence at Fentale-Dofen volcanoes, Ethiopia

**DOI:** 10.1007/s00445-025-01884-3

**Published:** 2025-10-12

**Authors:** Lin Way, Juliet Biggs, Weiyu Zheng, Milan Lazecky, Edna W. Dualeh, Tim Wright, Raphaël Grandin, Arthur Hauck, Sue Loughlin, Julia Crummy, Elias Lewi

**Affiliations:** 1 COMET, School of Earth Sciences, University of Bristol, Bristol, UK; 2 COMET, School of Earth and Environment, University of Leeds, Leeds, UK; 3 Institut de Physique du Globe de Paris, Université Paris Cité, 75005 Paris, France; 4 British Geological Survey, Edinburgh, UK; 5 Institute of Geophysics, Space Science and Astronomy, Addis Ababa University, Addis Ababa, Ethiopia

## Introduction

Seismic activity near Fentale volcano in the Main Ethiopian Rift started in late September 2024 and a M4.9 earthquake on 27th September was felt as far away as Addis Ababa (120 km) (Lewi et al. 2025). As ground access to part of the affected region was not possible, Addis Ababa University contacted several international organisations, including the UK Centre for the Observation and Modelling of Earthquakes, Volcanoes and Tectonics (COMET) to assist in analysing InSAR satellite data to help with the response. COMET responded by providing satellite observations of ground deformation and surface changes, and preliminary interpretations.

Over the next 6 months (September 2024 to March 2025), a sequence of magmatic dyke intrusions occurred within the Fentale-Dofen magmatic segment, accompanied by activity within the caldera of Fentale. The largest of these intrusions was ~ 50 km in length causing ~ 3 m of surface displacement; it lasted 60 days (17 Dec 2024–15 Feb 2025). The proximity of nearby towns, villages and the Ethiopia-Djibouti road and railway connections to the site of magma intrusion generated concern in the face of increasing activity (Lewi et al. 2025). The United Nations Office for the Coordination of Humanitarian Affairs (OCHA) issued a report on 23rd January stating that ~ 75,000 people were being evacuated (UN OCHA 2025).

Throughout the crisis, COMET’s real-time analysis of the satellite data was shared with our partners at Addis Ababa University and used as one of the inputs by a scientific committee comprising scientists from Addis Ababa University (IGSSA and School of Earth Science), the Geological Institute of Ethiopia and other relevant institutions to monitor the events and keep the Ethiopian Disaster Risk Management Commission (EDRMC) and the public continuously informed (Lewi et al. 2025). COMET also published a series of Event Response Reports through the COMET website (https://comet.nerc.ac.uk/publications/event-response-reports/) to support situational awareness and decision making by relevant stakeholders. These reports supported the British Geological Survey (BGS) International Natural Hazards Forward Look (INHFL) reports and volcano advisory assessments for the UK’s Foreign, Commonwealth and Development Office (FCDO). Satellite observations played a critical role in informing and supporting crisis response efforts.

This data report details the observations that were originally contained in COMET Event Response Reports 1.1–1.8 published online, covering the period from 12th September 2024–11th March 2025. Section 3 covers the first intrusion from September – October 2024, summarising Event Response Reports 1.1–1.2 published online on 8th and 22nd October respectively. Section 4 covers the subsequent intrusions from 19th December 2024–11th March 2025, and is a summary of Event Response Reports 1.3–1.8 published online on 6th, 14th, 29th January, 5th, 18th February and 13th March respectively. Section 5 focuses on observations of activity within Fentale caldera starting on 14th January, that were originally contained in COMET Event Response Reports 1.5–1.7. The Event Response Reports were independently reviewed by Sue Loughlin and Julia Crummy of the BGS and Elias Lewi of Addis Ababa University.

## Methodological approach

COMET’s response was underpinned by our national capability datasets and services, and long-term partnerships. Datasets used include:

- InSAR images collected by the European Sentinel-1 satellite and processed in near-real time using the automated COMET LiCSAR system (Lazecký et al. 2020), and timeseries from the European Sentinel-1 satellite archive spanning 2014–2024 and processed using the COMET LiCSBAS time series analysis tool (Morishita et al. 2020). Both the images and timeseries are provided open access through the COMET Volcanic and Magmatic Deformation Portal (https://comet.nerc.ac.uk/comet-volcano-portal/) (Watson et al. 2022).
- InSAR and SAR backscatter images acquired by the COSMO-SkyMed (CSK) and COSMO-SkyMed 2nd generation (CSG) satellites, provided through the CEOS GVEWERS programme and processed at the University of Bristol using GAMMA. Available here 10.5281/zenodo.16995116*.* Note that there is significant noise and decorrelation in several CSK interferograms due to a combination of atmospheric phase delays and large spatial baselines, which do not represent ground deformation.
- Digital Surface Model (DSM) based on the DSM-OPT webservice (https://en.poleterresolide.fr/dsm-opt-service/) developed and operated by FormaTerre, Solid Earth component of the Data Terra Research Infrastructure. Pléiades images were provided under the CIEST^2^ (CNES-FormaTerre) initiative (image Pléiades ©CNES2025, distribution AIRBUS DS). This dataset is licenced under CC BY-NC 4.0.
- High-resolution optical imagery (3 m pixel size) collected daily by the PlanetScope satellites of the Planet Lab constellation (Planet, 2025). Grids of estimated horizontal (east–west and north–south) components of ground displacement are available from Hauck, Grandin, Delorme (2025) (10.57932/b4ce1bad-a61b-499a-a3aa-36b632f45e56)
- Copernicus Sentinel-2 imagery (https://browser.dataspace.copernicus.eu/)
- USGS Earthquake Catalogue (https://earthquake.usgs.gov/earthquakes/search/)

## September–October 2024 unrest

### Event Response Report 1.1 (12th September–8th October 2024)

**Fig. 1:**
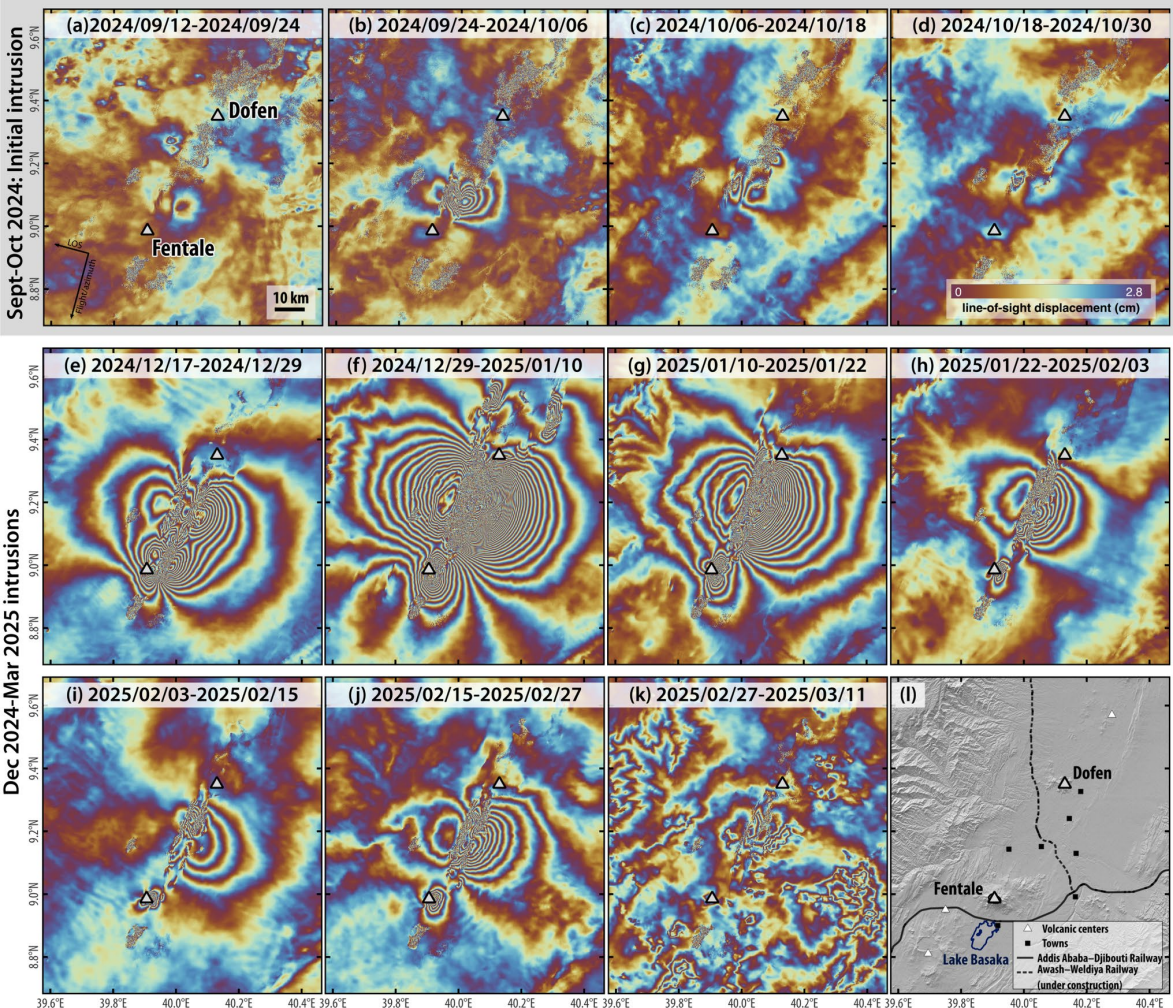
Twelve-day Sentinel-1 descending interferograms processed using the automated COMET LiCSAR system covering the initial September–October intrusion and subsequent intrusions from mid-December 2024–mid-March 2025. Ascending interferograms are shown in Supplementary Fig. S1. Note that there is significant noise in several interferograms (e.g., Fig. 1f–h, k) due to atmospheric phase delays which do not represent ground deformation

The first COMET Event Response Report (1.1) was posted on 8th October 2024 and covered the period 12th September—8th October 2024. A Sentinel-1 InSAR image between 24th September and 6th October (Fig. 1b) showed a butterfly deformation pattern typical of dyke intrusions: lateral motion in two outer lobes and subsidence in the centre. Visual inspection of the deformation pattern showed that the intrusion occurred about 13.6 km NE of Fentale volcano in the area known as Tinishu Fentale (centre 9.084N; 39.980E). The lateral extent of the deformation was ~ 15 km with a maximum displacement of ~ 17 cm in the satellite line-of-sight direction. The intrusion was probably associated with three M4-5 earthquakes that occurred on 27th September (USGS Catalogue). There was some evidence of surface faulting, but no sign of any eruption. The Sentinel-1 InSAR interferogram from 12th-24th September (Fig. 1a) suggests that the dyke intrusion started before September 24th, causing up to 3 cm of deformation but without any globally detected earthquakes. A M4.9 earthquake on 6th October occurred just after the satellite image was acquired. We noted that the area had experienced previous seismic swarms, including a dyke intrusion in 2015 that caused about 5 cm of deformation and an earthquake swarm with magnitudes up to 4.3 but did not result in an eruption (Temtime et al 2020; Ayele et al 2024).

### Event Response Report 1.2 (8th–22th October 2024)

**Fig. 2:**
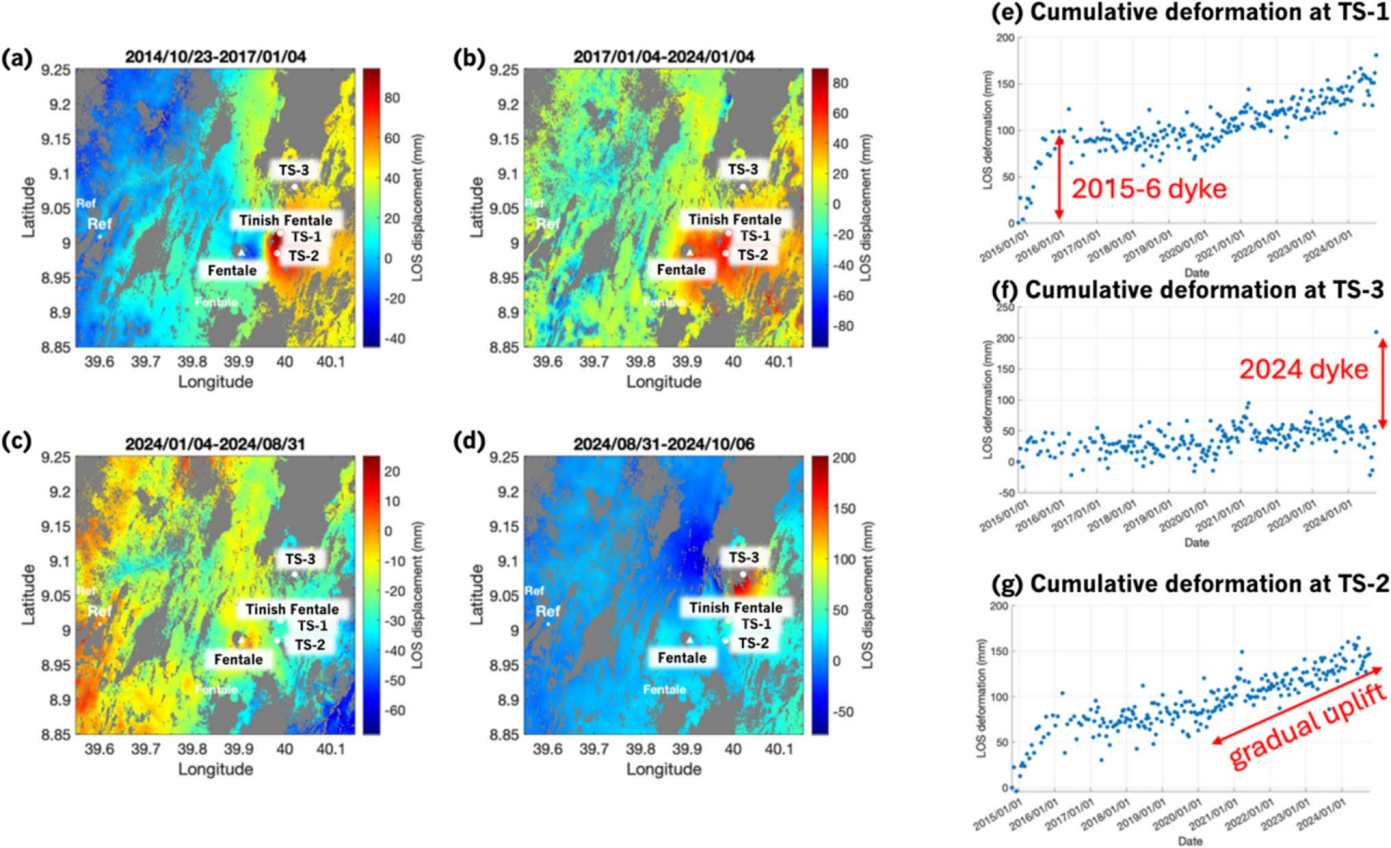
Sentinel-1 time-series from 2014 to 2024 processed using the COMET LiCS system (Lazecký et al. 2020; Morishita et al. 2020). **a** 23 October 2014–4 January 2017 showing the 2015 dyke intrusion previously reported by Temtime et al. (2020) and Ayele et al. (2024); **b** 4 January 2017–4 January 2024 showing slow uplift between Fentale and Tinish Fentale; **c** 4 January 2024–31 August 2024 showing no significant deformation prior to the activity in Sept 2024; **d** 31 August 2024–6 October 2024 showing the start of the September–October dyke intrusion. **e**, **f**, **g** Time series results at TS1, TS3, and TS2, respectively

Event Response Report 1.2 was posted on 22nd October 2024 and covered the period 8th-22nd October 2024. The latest Sentinel-1 InSAR image (6th-18th Oct; Fig. 1c) showed that the rate of deformation had decreased to ~ 6 cm from ~ 17 cm in the previous image (24th Sept-6th Oct; Fig. 1b), with a slight shift to the north (~ 3 km). The pattern of deformation was consistent with further opening of the dyke and slip along two inward dipping faults which ruptured the surface. As robust modelling of the deformation was not possible in real-time during the response, we refer the reader to Keir et al. (2025) which details geodetic modelling results of dyke and fault parameters during the period from 12 September to 11 November. COSMO-SkyMed images provided additional constraints on the timing of deformation and indicated that little deformation took place between 3rd-12th October (Fig. S2). The USGS reported 3 additional M4 + earthquakes in this time period, on 6th, 13th and 16th October. Processing of the entire Sentinel-1 archive showed: the 2015 dyke intrusion (Fig. 2a), slow uplift in 2017–2024 located between Fentale and Tinish Fentale (Fig. 2b) and the 2024 dyke, which is located north along strike of the 2015 dyke intrusion (Fig. 2d).

## December 2024–March 2025 intrusions

### Event Response Report 1.3 (22th October 2024–3rd January 2025)

Event Response Report 1.3 was posted on 6th January 2025 and covered the period 22nd October 2024–3rd January 2025. Following a period of relative quiescence after the September–October dyke intrusion, seismic activity restarted on 20th December (Institute of Geophysics, Addis Ababa University) (Lewi et al. 2025). The most recent 12-day Sentinel-1 InSAR image (17–29 December) showed a significantly larger surface deformation than the previous intrusion that occurred in September–October (Fig. 1e). The dyke intrusion was about 40 km long, extending from Fentale to just south of Dofen. The orientation of the dyke (NE-SW) was similar to both the previous intrusions in 2015 and in Sept-Oct 2024, but it extended further north and south. In total, there was ~ 33 cm of displacement in the satellite line-of-sight, almost twice that of the ~ 17 cm displacement in September–October. As with the previous interferograms, there was evidence of surface fault rupture. The deformation pattern suggested variations in the magnitude of dyke opening corresponding to distinct lobes of deformation, as well as a possible deflating source beneath Fentale.

### Event Response Report 1.4 (5th–10th January 2025)

Event Response Report 1.4 was posted on 14th January 2025 and covered the period 5th-10th January 2025. At the time of writing, unrest was ongoing, with the latest 12-day Sentinel-1 descending interferogram showing further northeast-wards propagation of the dyke (~ 8 km longer), where deformation reached Dofen (Fig. 1f). Maximum line-of-sight (LOS) surface displacement associated with dyke opening was larger than 1 m. Subsidence at Fentale had also increased, from ~ 28 cm of LOS displacement away from the satellite from 17th-29th December to ~ 45 cm of LOS displacement from 29th December – 10th January. Deformation visible to the north of Dofen is likely associated with surface faulting due to the > M5 earthquakes reported by the USGS.

**Fig. 3:**
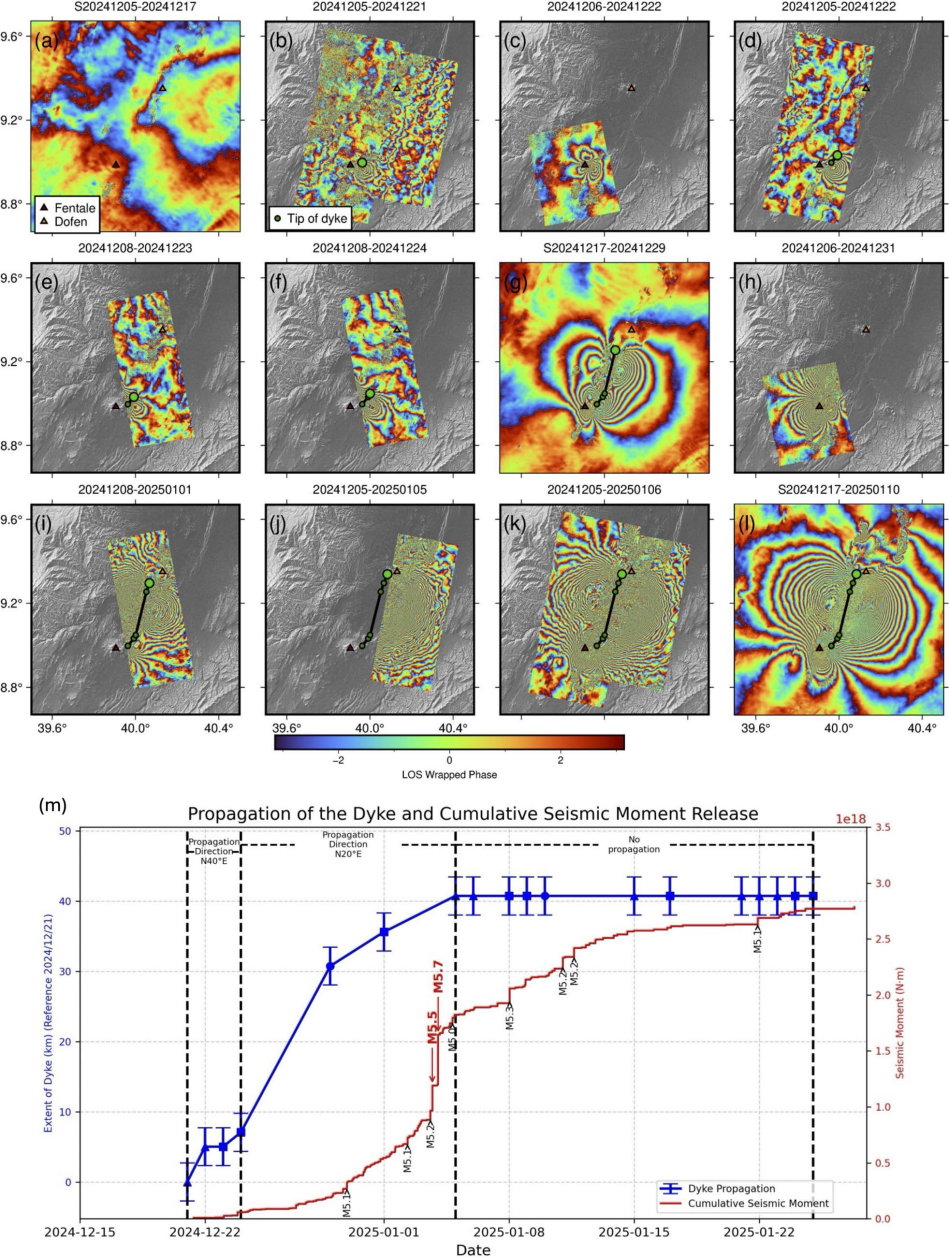
**a**–**l** Wrapped interferograms from COSMO-SkyMed (CSK) and Sentinel-1 (labelled with prefix ‘S’). For Sentinel-1 interferograms, each fringe corresponds to ~ 2.8 cm of displacement in the satellite line of sight, while each fringe in COSMO-SkyMed interferograms correspond to ~ 1.6 cm of displacement in the line of sight. **m** Extent of dyke (blue line) and cumulative seismic moment release (red line) over time (updated from Event Report 1.4), since the start of the December 2024 unrest. Note that there is significant noise and decorrelation in the CSK interferograms due to a combination of atmospheric phase delays and large spatial baselines, which do not represent ground deformation

The temporally dense acquisitions of COSMO-SkyMed (CSK) InSAR data enabled monitoring of the dyke propagation. Figure 3 presents wrapped interferograms from CSK (ascending and descending tracks) and Sentinel-1 data, spanning the period from 5th December to 10th January. The start date precedes the onset of the event, and the wrapped interferograms are plotted in chronological order to show the progression of the dyke intrusion. Figure 3m illustrates the progression of the dyke and clearly show the effect that segmentation and change in orientation have on the rate of tip propagation:

- Deformation began sometime between 17th and 21 st December (Fig. 3a,b).
- Initially, deformation was localised within 25 km of Fentale, with deformation up to approximately 15.6 cm in the LOS direction (Fig. 3b–f). During this time, the dyke tip propagated about 7 km in 3 days at a bearing of ~ 040 degrees.
- Between 24 and 29th December, there was a change in direction, accompanied by an acceleration in dyke propagation. The dyke tip propagated about 24 km in 5 days (Fig. 3g) and reached the neighbouring volcano Dofen, 47 km to the north of Fentale. The direction of the dyke changes from radial to Fentale to aligned with the rift axis (N20°E). LOS deformation associated with dyke opening is ~ 36 cm.
- After 29th December, the dyke continued to propagate but at a slower rate of about 15 km in 12 days (Fig. 3g–l). The total LOS deformation reached > 1 m by 10th January, which corresponds to significant dyke opening without significant tip propagation.
- The ascending track of the CSK data (Fig. 3c,h) shows continuous subsidence at Fentale, with local LOS displacement reaching up to 36 cm on 31 st December.

### Event Response Report 1.5 (10th–25th January 2025)

COMET Event Response Report 1.5 was posted on 29th January 2025 and covered the period 10th-25th January 2025. Sentinel-1 and COSMO-SkyMed (CSK) interferograms showed continued unrest and deformation at a slower rate. The latest 12-day descending Sentinel-1 interferogram suggested that the rate of subsidence at Fentale and dyke opening had slowed when compared to the previous 12-day interferogram. Line-of-sight (LOS) displacement away from the satellite at Fentale decreased from ~ 45 cm during 29th December 2024–10th January 2025 to ~ 33 cm during 10th-22nd January 2025. Deformation associated with dyke opening had also decreased, from > 1 m in the previous 12-day period, to > 65 cm in the most recent 12-day period (Fig. 1g). The dyke has neither propagated further northwards, nor was there a change in propagation direction.

Ascending and descending CSK interferograms with identical temporal baselines (8-day descending and 1-day ascending) also suggested a slowing deformation at Fentale and dyke opening (Fig. S3). From the 8-day descending images, the LOS displacement away from the satellite at Fentale decreased from > 25 cm (7th-15th January) to > 11 cm (15th-23rd January) (Fig. S3a-b). The 1-day ascending images showed a decrease in deformation related to dyke opening from ~ 12 cm LOS displacement from 8th-9th Jan to ~ 3 cm from 24th-25th January (Fig. S3d, g). The dyke opening was now localised to the northern segment, with the length of the dyke opening decreasing from ~ 35 km to ~ 13 km, from the most recent 1-day interferogram (Fig. S3g).

Subsidence at Fentale was ongoing, but the deformation pattern was changing based on consecutive CSK interferograms (Fig. S4). Earlier on during the December 2024 unrest, a simple deflating source beneath Fentale might be able to explain majority of the deformation. However, more recent deformation patterns suggest that additional sources are required. The latest images point towards the likelihood of several NE-SW trending linear features (possibly faults and/or dykes) located north and northeast of Fentale that are increasingly dominating the deformation signal (Fig. S4a,d).

### Event Response Report 1.6 (22nd January–3rd February 2025)

COMET Event Response Report 1.6 was posted on 29th January 2025 and covered the period 22nd January – 3rd February 2025. At the time of writing, unrest was ongoing, but continuing at a slower rate than December 2024. Based on the latest 12-day descending Sentinel-1 interferogram spanning 22nd January to 3rd February, line-of-sight (LOS) displacement associated with dyke opening was > 20 cm. In comparison, LOS displacement was > 65 cm from 10th-22nd January, and > 1 m from 29th December to 10th January (Fig. 1f–h). At Fentale, displacement away from the satellite was ~ 14 cm. This is a decrease from the ~ 33 cm observed during the previous 12-day period from 10th-22nd January, and ~ 45 cm from 29th December – 10th January. Event Report 1.5 stated that the dyke opening was localised to a 13 km long segment just south of Dofen, based on a COSMO-SkyMed (CSK) interferogram spanning 24th-25th January. However, a more recent interferogram from 25th January to 2nd February shows opening along a ~ 33 km long dyke (Fig. S3h). This highlights that the rate of opening is variable throughout the entire length of the dyke, and caution should be exercised when interpreting short-temporal baseline interferograms.

### Event Response Report 1.7 (14th January–16th February 2025)

COMET Event Response Report 1.7 was posted on 18th February 2025 and covered the period 14th January—16th February 2025. Most of the report was related to activity within Fentale caldera, which is described in Section 5. The latest 12-day ascending and descending Sentinel-1 interferograms (3rd-15th February) continued to show a decrease in deformation rate associated with dyke opening, evidenced by the reduced number of fringes within consecutive 12-day intervals (Fig. 1g–i). There was > 8 cm of LOS displacement from 3rd-15th February, which is a decrease from > 20 cm from 22nd January – 3rd February, and > 65 cm from 10th-22nd January.

### Event Response Report 1.8 (15th February–11th March 2025)

COMET Event Response Report 1.8 was posted on 13th March 2025 and covered the period 15th February – 11th March 2025. We had previously reported a slowdown in dyke opening based on Sentinel-1 images acquired on 15th February (in Event Response Report 1.7). Since then, there has been another pulse of magma intrusion, with an increase in dyke opening (~ 25 cm LOS displacement) observed between 15th-27th February before slowing down in the following 12-day period from 27th February to 11th March with ~ 5 cm LOS displacement (Fig. 1j,k). This was accompanied by a correlated increase in subsidence beneath Fentale and subsequent decrease. This pulse (reopening over ~ 12 days) was much shorter than the previous one which lasted approximately from 17th December 2024 to 15th February 2025.

## Activity within Fentale caldera: Event Response Reports 1.5–1.7 (10th January–16th February 2025)

Event report 1.5 posted on 29th January 2025, which covered the period 10th-25th January 2025, reported that thermal anomalies and plumes had been observed at Fentale from infrared (MIROVA detections of hotspots from MODIS sensors) and optical (e.g., Sentinel-2) satellites since mid-January. Event Report 1.6 posted on 5th February and covering the period 22nd January – 3rd February 2025 reported that plumes within the crater of Fentale continued to be visible in satellite optical imagery (e.g., Sentinel-2).

On 7th February 2025, a news story on the GHGSat website reported that substantial emissions of methane had been detected around Fentale using TROPOMI since 19th January, with rates of 58 tonnes/hr detected using GHGSat from within Fentale caldera on 31 st January (GHGSat 2025). No carbon dioxide or sulfur dioxide emissions were reported at the time of writing. In Event Response Report 1.7 posted on 18th February 2025, we reprocessed the previously reported satellite datasets, using a high-resolution Digital Surface Model (DSM) of Fentale derived from Pléiades images, resulting in a pixel size of 2 m.

**Fig. 4:**
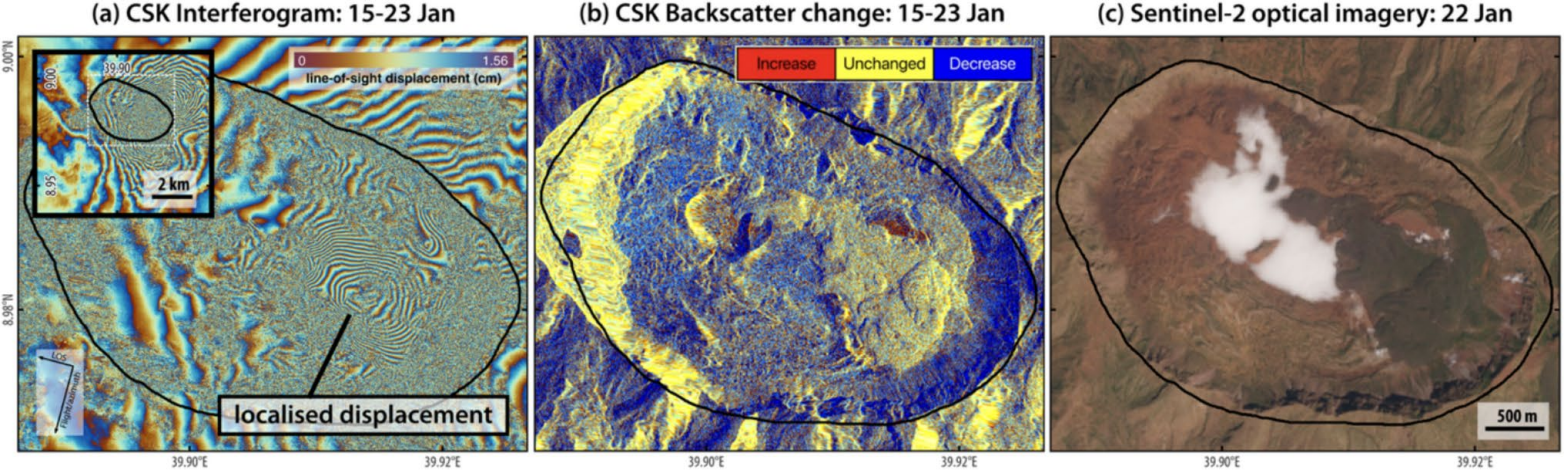
Satellite SAR and optical data acquired between 15 and 23 Jan after the first detection of thermal anomalies within Fentale caldera by MIROVA on 14 Jan 2025. **a** CSK descending interferogram showing broader subsidence extending past the caldera (inset) and localised crater subsidence of ~ 31 cm away from the satellite from 15 to 23 Jan. **b** Changes in SAR backscatter could be due to increase in moisture content between 15 and 23 Jan. **c** White plumes visible in Sentinel-2 optical imagery on 22 Jan

Reprocessed COSMO-SkyMed interferograms spanning 15th-23rd January with a higher resolution DSM showed localised deformation (predominantly subsidence) within the caldera, with line-of-sight (LOS) displacement away from the satellite of ~ 31 cm (Fig. 4a). This deformation occurred over a small spatial scale, and it was not possible to resolve it using the original data processed at 10 m using an oversampled 30 m SRTM DEM. The overlapping deformation patterns, with broader subsidence across more than 15 km and localised intra-crater subsidence over ~ 2 km, suggested there were likely to be multiple deformation sources (Fig. 4a). We proposed that methane-rich gases were stored in the shallow subsurface at least temporarily prior to emission.

COSMO-SkyMed SAR images showed an increase in backscatter northeast to the localised subsidence that could be related to increase in moisture content in the ground (Fig. 4b). The location of backscatter changes correlated with where plumes were observed (Fig. 4c). Hydrothermal alteration within the caldera was also visible since mid-January. This coincides with an apparent degradation of vegetation. However, due to lack of direct in situ observations, it remains unclear whether dry meteorological conditions prevailing in the same period may have been the primary factor driving this decay, or if enhanced hydrothermal activity may have also played a role.

### 14th February 2025: M 6.0 earthquake and localised collapse

**Fig. 5:**
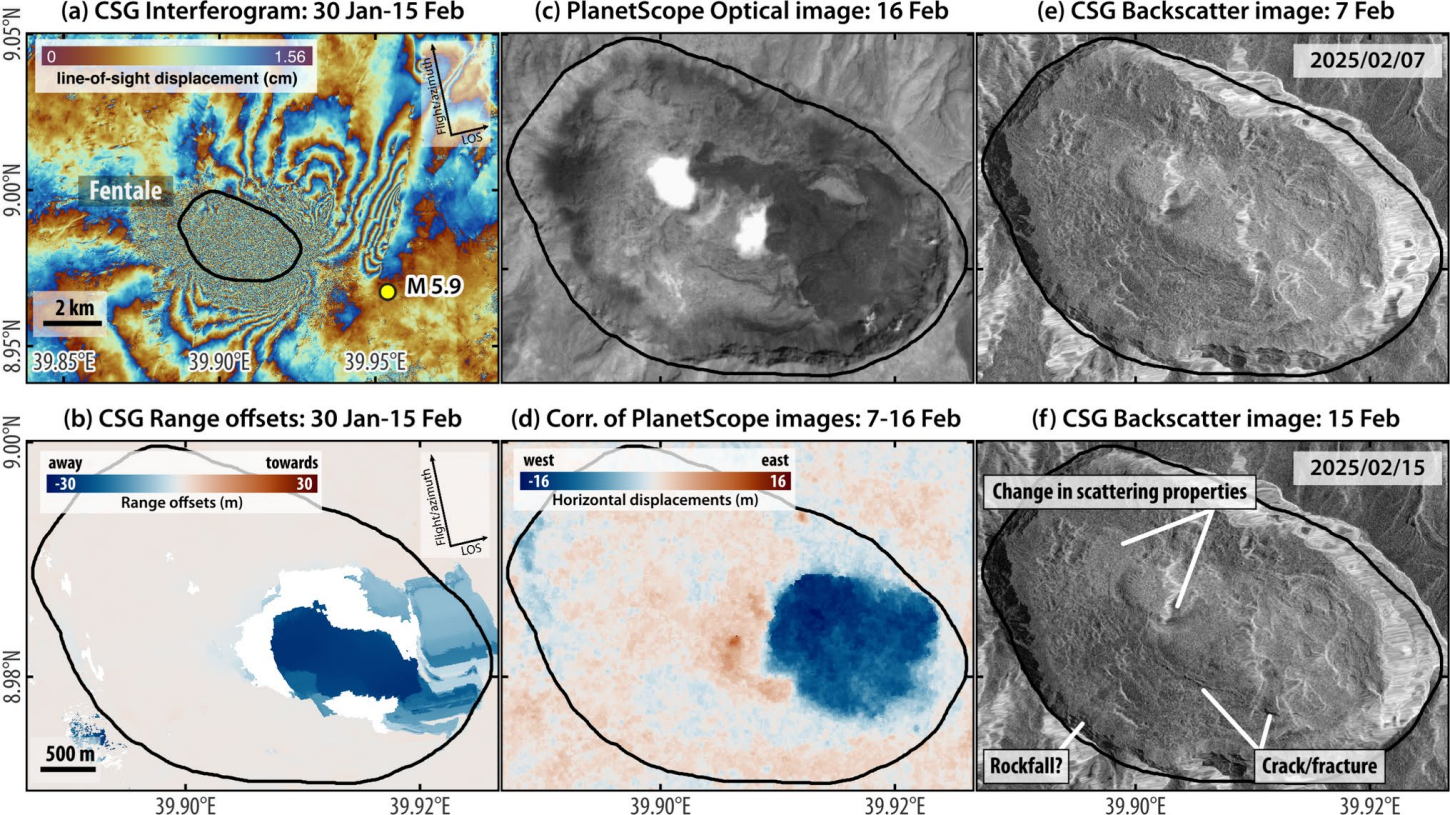
Satellite SAR and optical data spanning the M5.9 earthquake recorded on 14 February 2025, UTC 20:28. **a** COSMO-SkyMed 2nd generation (CSG) interferogram and **b** range offsets showing motion away (blue) from the satellite of ~ 30 m between 30 January and 15 February. **c** PlanetScope panchromatic image acquired on 16 February, showing at least two sites of steam emissions. **d** Horizontal surface displacement from correlation of PlanetScope images acquired on 7 and 16 Feb (pixel size: 3 m), where negative values (in blue) indicate motion toward the west (Hauck et al. 2025). The correlation was done using the MicMac software (Rosu et al. 2015). Imagery © 2025 Planet Labs Inc. **e**, **f** CSG backscatter images show changes in ground scattering properties that might be related to possible rockfall and crack formations

On 14th February UTC 20:28, an earthquake with magnitude 5.9–6.0 was reported by the USGS. The focal mechanism was a vertical-P compensated-linear-vector-dipole (CLVD) that could not be explained by shear along planar faults associated with pure double-couple forces. In volcanic settings, CLVD earthquakes have been attributed to dip slip motion along curved ring faults (Shuler et al. 2013). For example, similar mechanisms had previously been documented at Bárdarbunga volcano, Iceland in 1996 and during the 2014–2015 caldera collapse (Gudmundsson et al. 2016; Nettles and Ekström 1998), and during the co-diking caldera ring faulting at Ambrym volcano, Vanuatu, in 2018 (Shreve et al. 2019). Preliminary investigation of Sentinel-1 and CSG InSAR images available at the time of writing spanning this earthquake did not show conclusive evidence of ring faulting within Fentale caldera. However, normal sense slip occurred along a ~ 4 km fault located about 5 km east of the centre of the crater (Fig. 5a).

Within the caldera, SAR range offsets showed motion of up to ~ 30 m away from the satellite which could be representative of slumping (Fig. 5b). Concurrently, in the same area, Planet imagery showed up to ~ 15 m of horizontal displacement toward the west (Fig. 5d). These large displacements could represent a local collapse of material due to volume loss at shallow depth. Deformation along the eastern wall of the caldera is visible in satellite optical imagery from Sentinel-2 and Planet after 14th February, and is likely associated with the M 6.0 earthquake. Changes in ground backscatter properties observed in CSG SAR backscatter images are suggestive of formation of cracks or fractures, and a possible rockfall (Fig. 5f).

## Conclusion

The satellite data reported here document the intrusion of a ~ 50 km long dyke between Fentale and Dofen volcanoes in two phases: Sept-Oct 2024 and Dec 2024-Mar 2025, with ongoing activity within Fentale caldera. The information presented here was used as one of the inputs by the Ethiopian Scientific Advisory Committee to keep the Ethiopian Disaster Risk Management Commission (EDRMC) and the public continuously informed (Lewi et al. 2025). It was also used by UK and international governmental organisations for situational awareness.

Although deformation and seismicity associated with the dyke intrusion is low at the time of writing (June 2025), many dyke intrusions occur in sequences lasting 7–10 years, including periods of quiescence lasting several months (Wright et al. 2012). We also noted that there are many examples where eruptions have occurred several months after a dyke intrusion, including the 2017 eruption of Agung, Indonesia (Albino et al 2019) and the 2007 eruption of Oldoinyo Lengai, Tanzania (Biggs et al 2009). At Agung and Oldoinyo Lengai, the eruption occurred from a volcanic edifice > 10 km from the dyke intrusion. This is particularly relevant because thermal anomalies and gas plumes have been observed at Fentale from infrared (MIROVA detections of hotspots from MODIS and VIIRS sensors) and optical (e.g., Sentinel-2) satellites since mid-January and continued to be visible in satellite imagery. We noted that the M 5.9–6.0 earthquake on 14th February, together with observations of slumping and subsidence within the caldera, could represent the beginning stages of a caldera collapse phase. The methane and carbon dioxide emissions were significantly larger than any previously reported from volcanoes (Etiope and Sherwood Lollar 2013).

We concluded that continued monitoring was critical to provide evidence on which the potential evolution of the event could be considered. We continued to monitor surface deformation with Sentinel-1 and COSMO-SkyMed images, and provide information to the Ethiopian Scientific Advisory Committee and UK government via the British Geological Survey. At the time of writing (June 2025), localised caldera deformation is continuing. As of 15th June 2025, thermal anomalies (detected by MIROVA using VIIRS) within the caldera of Fentale and plumes along the eastern wall of the caldera (Sentinel-2) continued to be visible in satellite imagery. This, in combination with other data, observations and models, can provide evidence on which the potential evolution of the event can be considered. Ground-based information such as GNSS deformation and earthquake catalogues (regional or local) can provide higher temporal resolution than satellite data. Of particular importance is understanding the deformation in the magma source zones as renewed uplift might precede further activity (Parks et al. 2025). For Fentale, this refers to both the area that uplifted in 2017–2024 (Fig. 2b) and the area that subsided during the January-March intrusion.

## Supplementary Information

Below is the link to the electronic supplementary material.

Supplementary file1 (DOCX 13.6 MB)

## Data Availability

All data reported here are publicly available without restriction. Sentinel-1 InSAR images can be accessed through the COMET Volcanic and Magmatic Deformation Portal (https://comet.nerc.ac.uk/comet-volcano-portal/). COSMO-SkyMed InSAR images are available here (10.5281/zenodo.16995116)*.* Copernicus Sentinel-2 imagery can be accessed through https://browser.dataspace.copernicus.eu/. Horizontal components of ground displacement are available from Hauck, Grandin, Delorme (2025) (10.57932/b4ce1bad-a61b-499a-a3aa-36b632f45e56*).* Access to Planet imagery is subject to license agreement, which can be requested from the @Planet company (Education and Research Standard Plan, contract ID 749508, PI Raphael Grandin). USGS earthquake catalogue is available from https://earthquake.usgs.gov/earthquakes/search/.
