# Supplementary material for: Real-time satellite monitoring of the 2024–2025 dyke intrusion sequence at Fentale-Dofen volcanoes, Ethiopia
Source: Bull Volcanol. 2025 Oct 12;87(11):100. doi: 10.1007/s00445-025-01884-3 (PMC12515745; doi:10.1007/s00445-025-01884-3)
Supplement: Supplementary file 1 — Supplementary file1 (DOCX 13.6 MB) [file 445_2025_1884_MOESM1_ESM.docx]

**Supplementary Information** *for Bulletin of Volcanology Data Report:*

Real-time Satellite Monitoring of the 2024-2025 dyke intrusion sequence at Fentale-Dofen Volcanoes, Ethiopia.

Lin Way^1*^, Juliet Biggs^1^, Milan Lazecky^2^, Weiyu Zheng^1^, Edna W. Dualeh^1^, Tim Wright^2^, Raphaël Grandin^3^, Arthur Hauck^3^, Sue Loughlin^4^, Julia Crummy^4^, Elias Lewi^5^

^1^ COMET, School of Earth Sciences, University of Bristol, Bristol, UK

^2^ COMET, School of Earth and Environment, University of Leeds, Leeds, UK

^3^ Université Paris Cité, Institut de physique du globe de Paris, 75005 Paris, France

^4^ British Geological Survey, Edinburgh, UK

^5^ Institute of Geophysics, Space Science and Astronomy, Addis Ababa University, Addis Ababa, Ethiopia

** Corresponding author: lin.way@bristol.ac.uk*

**
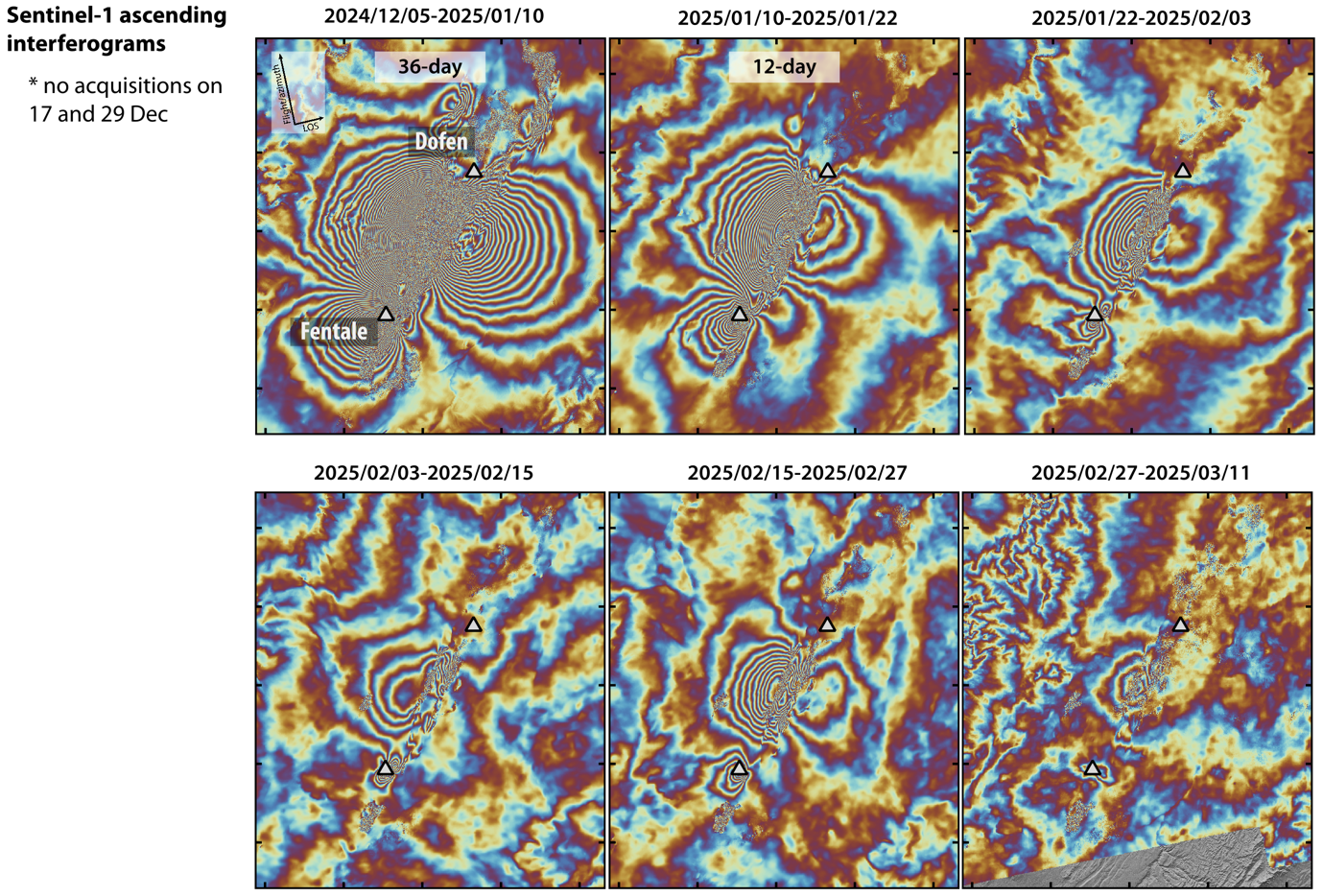
**

**Fig S1.** Sentinel-1 ascending interferograms processed using the automated COMET LiCSAR system covering the intrusions from mid-December 2024 – mid-March 2025. Note that the first panel is an interferogram spanning 36 days, while the other panels are 12-day interferograms.


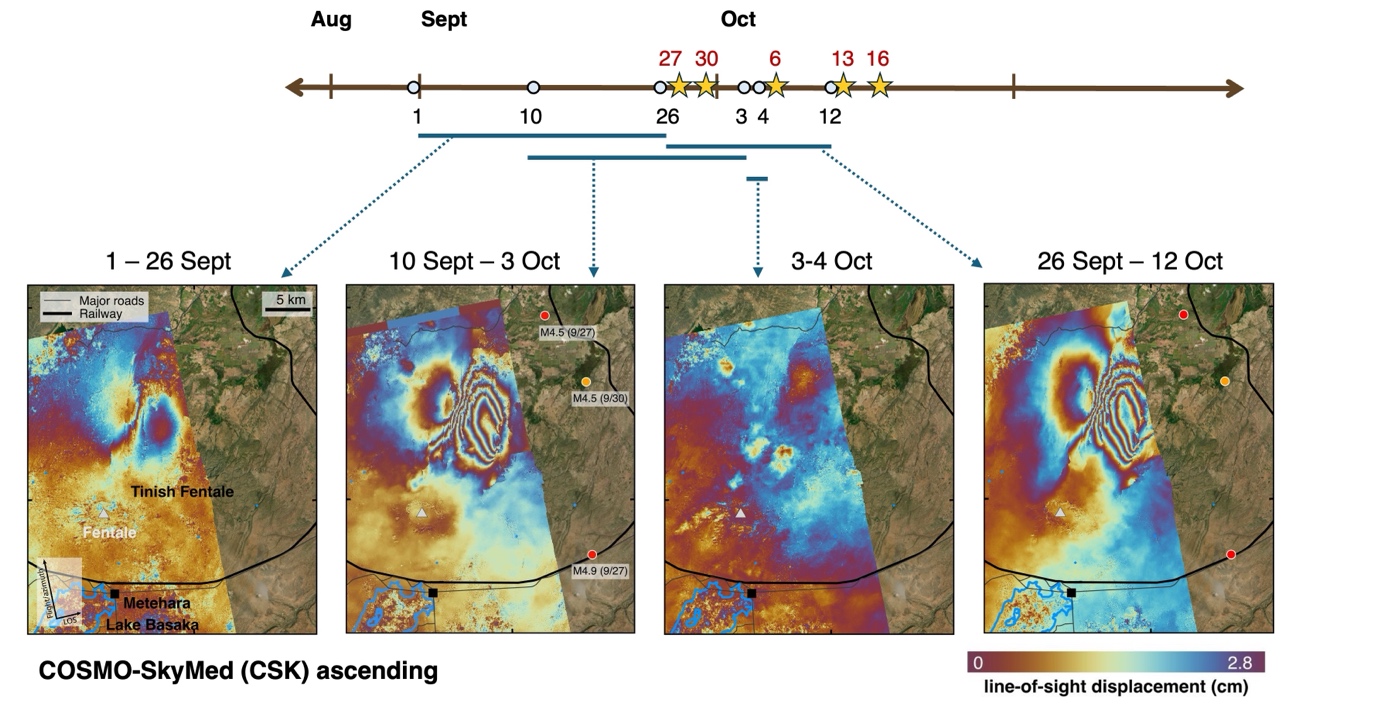


**Fig S2.** COSMO-SkyMed (CSK) images from 1^st^ Sept-12^th^ Oct 2024. Dates of CSK acquisitions are represented by white circles and reported >M4 earthquakes as yellow stars on the timeline. Note, the 10^th^ Sept -3^rd^ Oct image is almost identical to the 26^th^ Sept-12^th^ Oct image, suggesting little deformation took place between 3^rd^-12^th^ Oct. Originally published in COMET Event Response Report 1.2 (22 Oct 2024).


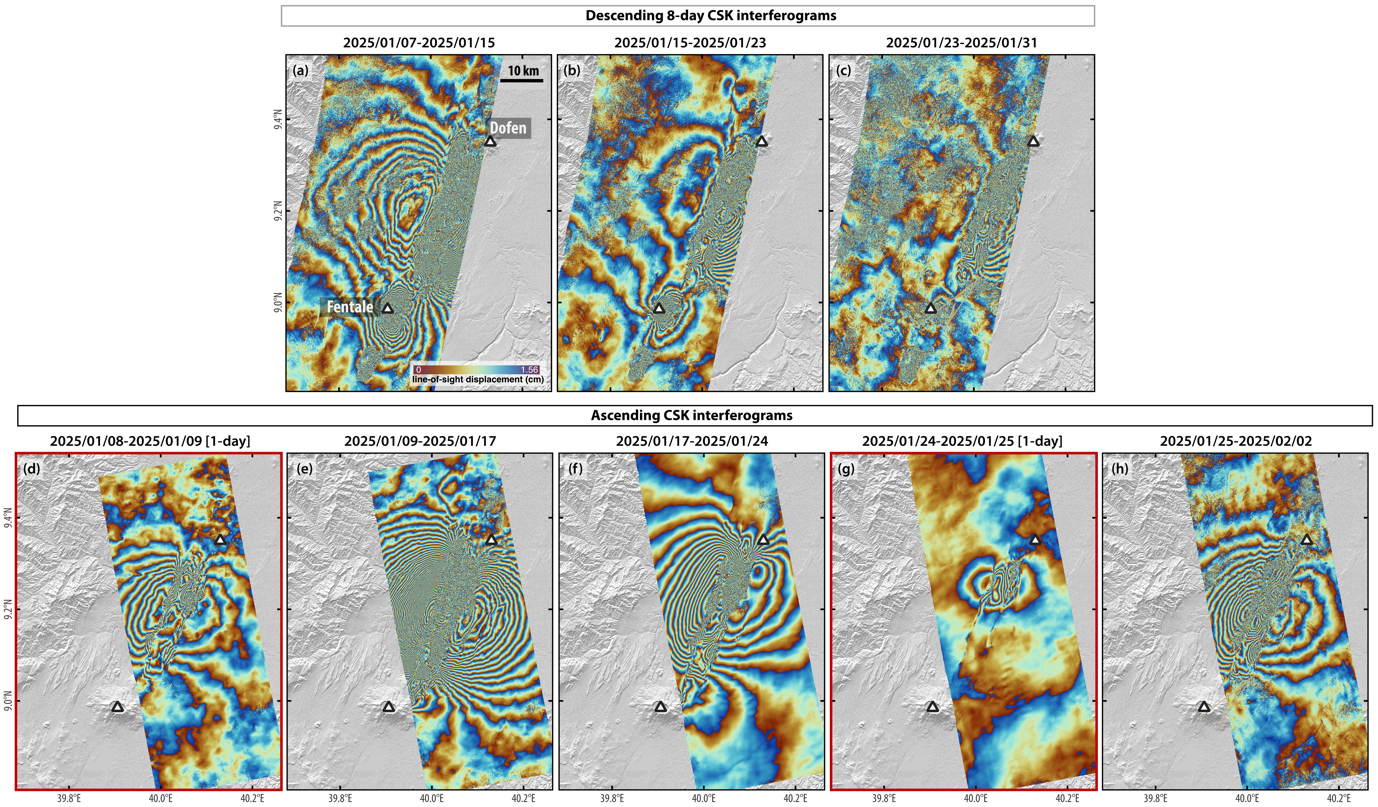


**Fig S3.** 8-day descending COSMO-SkyMed (CSK) interferograms (a-c) and 1-day ascending interferograms (d,g with red borders) showing a slowdown in dyke opening as well as subsidence at Fentale. The most recent 1-day interferogram (g) shows that the dyke opening is now localised within the northern segment, just south of Dofen.  However, the subsequent interferogram spanning 25^th^ January to 2^nd^ February (h) shows continued opening along a ~ 33 km dyke, suggesting that the rate of opening is variable along the length of the dyke. Originally published in COMET Event Response Report 1.5 (29 Jan 2025) and 1.6 (5 Feb 2025).


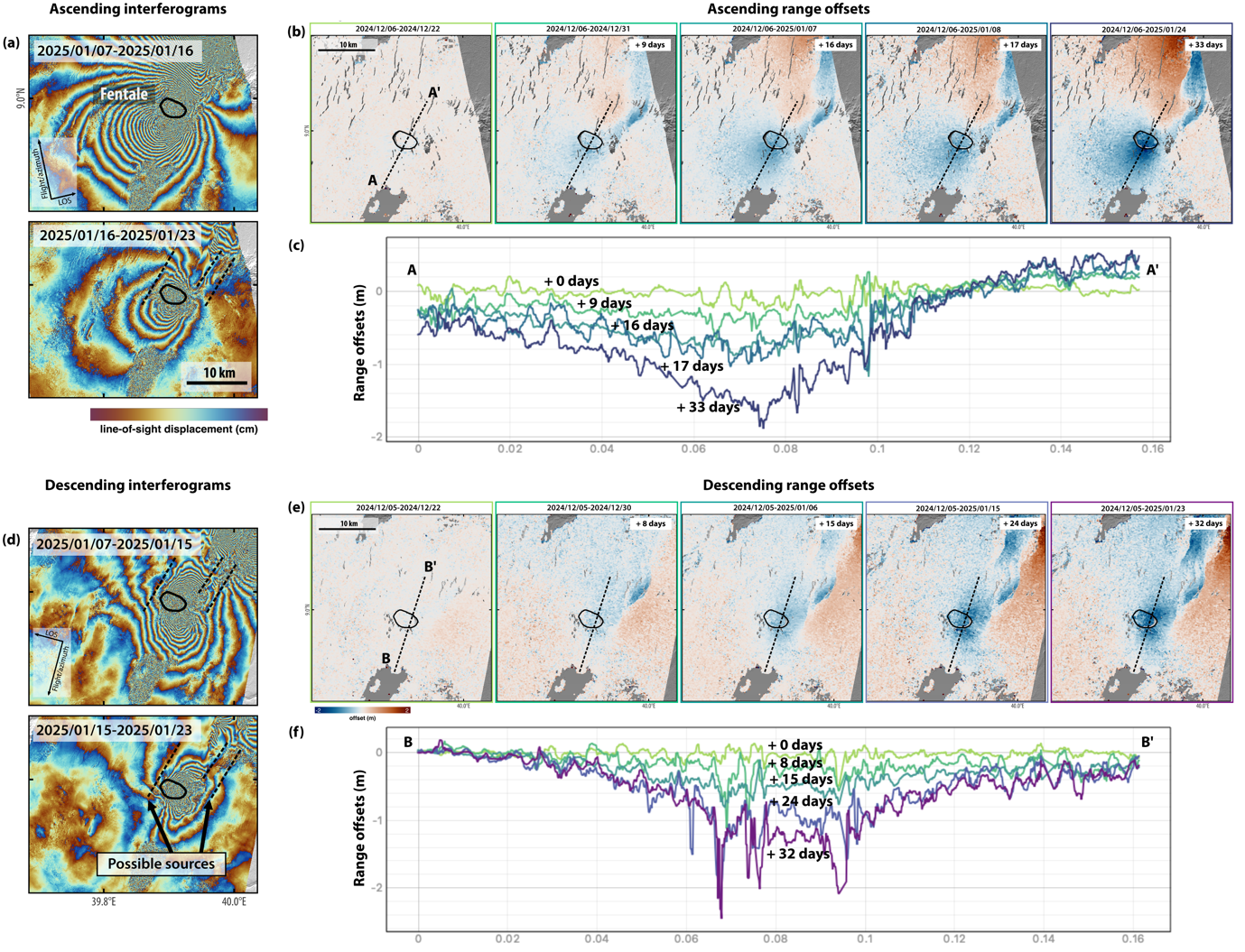


**Fig S4.** Change in deformation pattern at Fentale from the end of December 2024 to 24^th^ January 2025, shown by consecutive COSMO-SkyMed (CSK) ascending and descending interferograms centred on Fentale. Possible sources contributing to the deformation pattern change in the 16-23 Jan (a: ascending) and 15-23 Jan (d: descending) images are indicated by the dashed black lines with NE strike. The crater of Fentale is drawn in a black solid line. (b) Ascending and (e) descending cumulative CSK range offsets. Offsets are calculated with the same reference/starting date before the unrest started. (c, f) Profiles of range offsets across A-A’ and B-B’ do not show significant contributions from possible caldera ring faults along the longer axis of the caldera. Originally published in COMET Event Response Report 1.5 (29 Jan 2025).


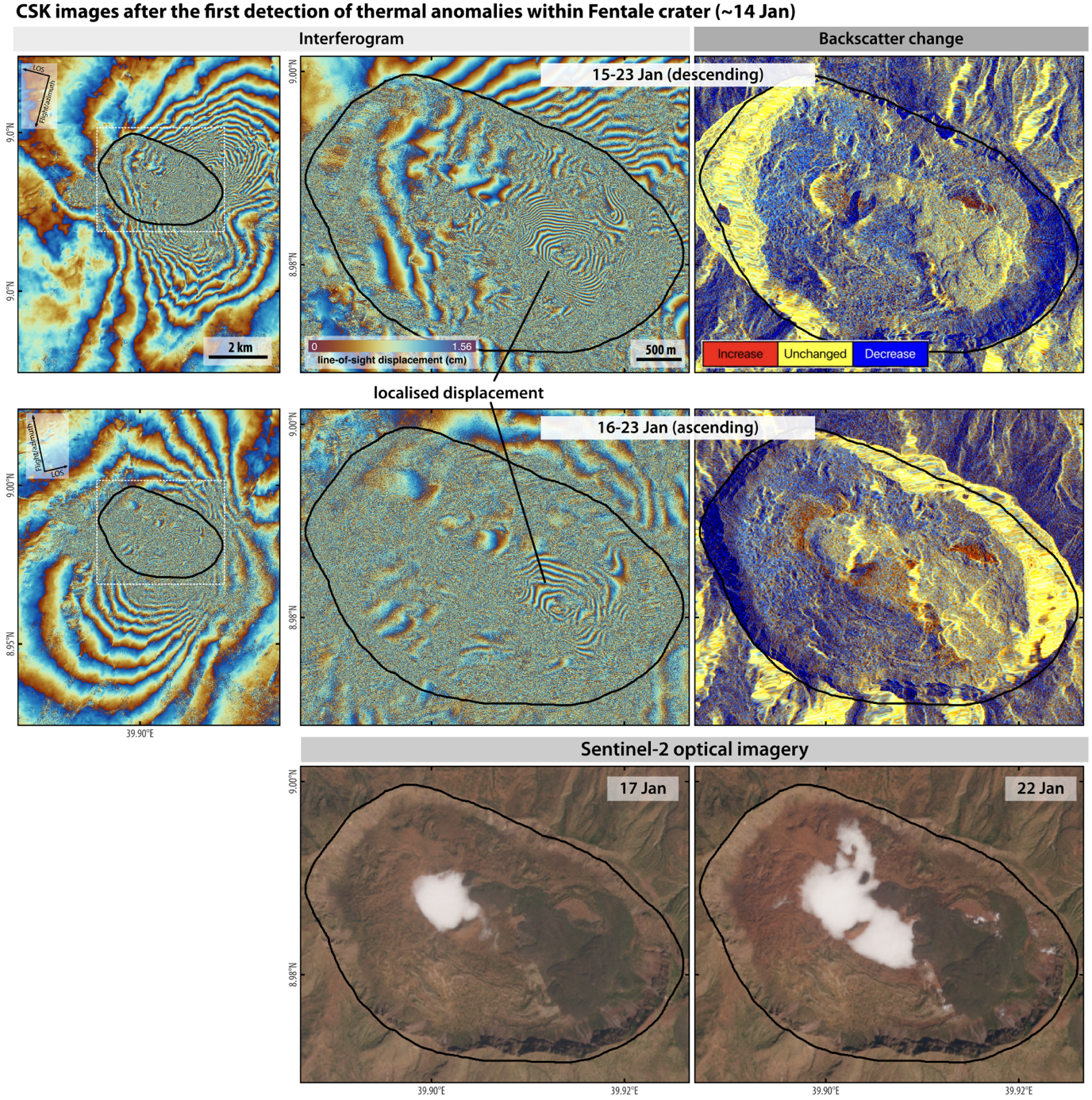


**Fig S5**. (Full extended Fig 4) COSMO-SkyMed interferograms showing localised deformation within Fentale crater, shortly after the first detections of thermal anomalies from VIIRS (MIROVA) on 14^th^ January and plumes from optical imagery (Sentinel-2 and Planet). There is approximately 31 cm of LOS displacement away from the satellite from 15^th^-23^rd^ January (descending acquisitions), and 14 cm from 16^th^-23^rd^ January (ascending). Changes in backscatter during the same time periods could be related to increased moisture content in the ground. Plumes are visible in the true colour Sentinel-2 optical imagery on 17^th^ and 22^nd^ January. Originally published in COMET Event Response Report 1.7 (18 Feb 2025).


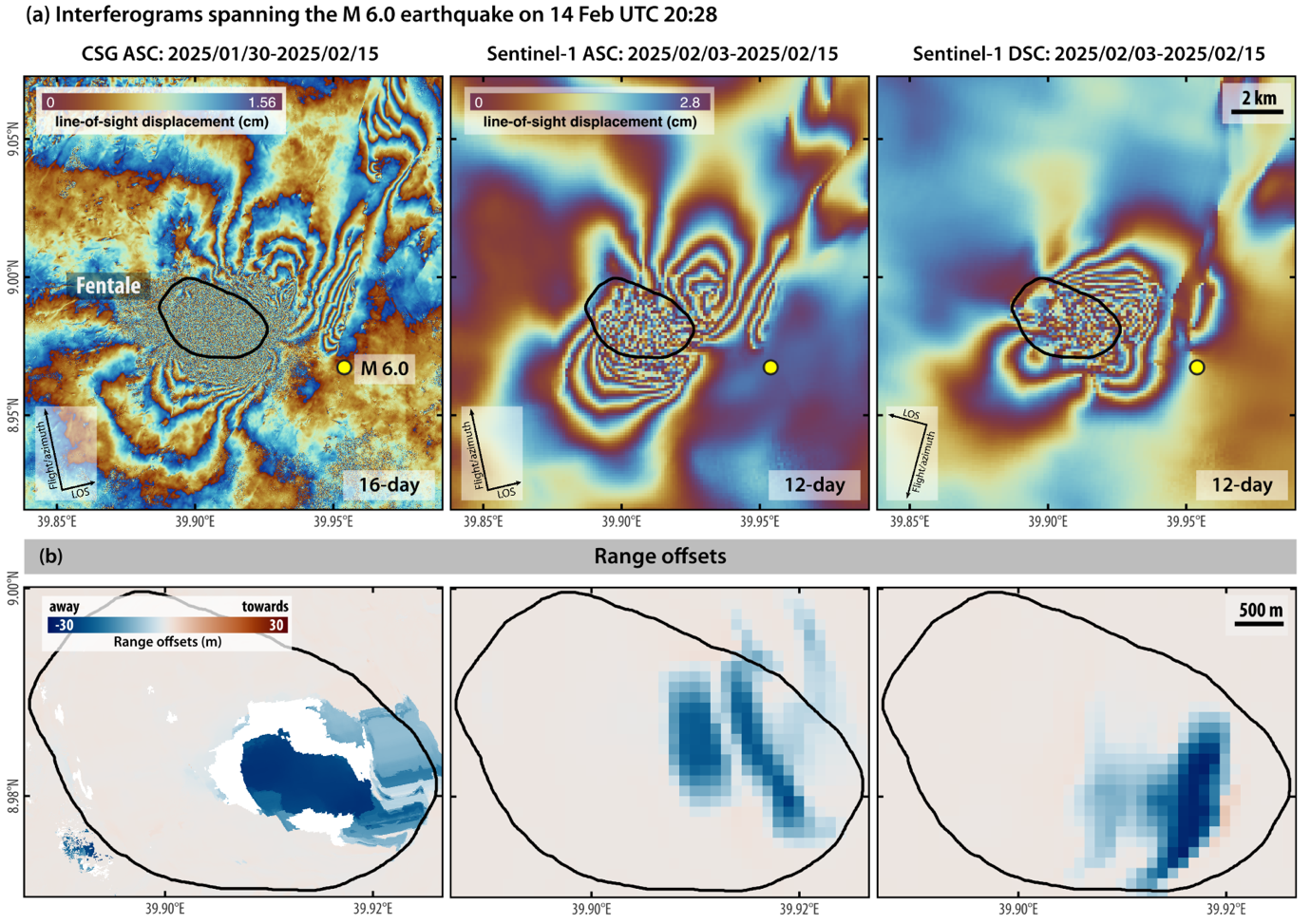


**Fig S6.** (Extended Fig 5 in main text) (a) 16-day ascending CSG and 12-day Sentinel-1 images centred on Fentale caldera, spanning the predominantly non-double-couple (vertical CLVD) M 6.0 earthquake recorded on 14^th^ February, UTC 20:28. Normal faulting is observed just north of the epicenter of the M 6.0 located by USGS. (b) Preliminary range offsets show motion away (blue) from the satellite of ~ 30 m. Areas with low SNR are masked in the CSG offsets. Originally published in COMET Event Response Report 1.7 (18 Feb 2025).


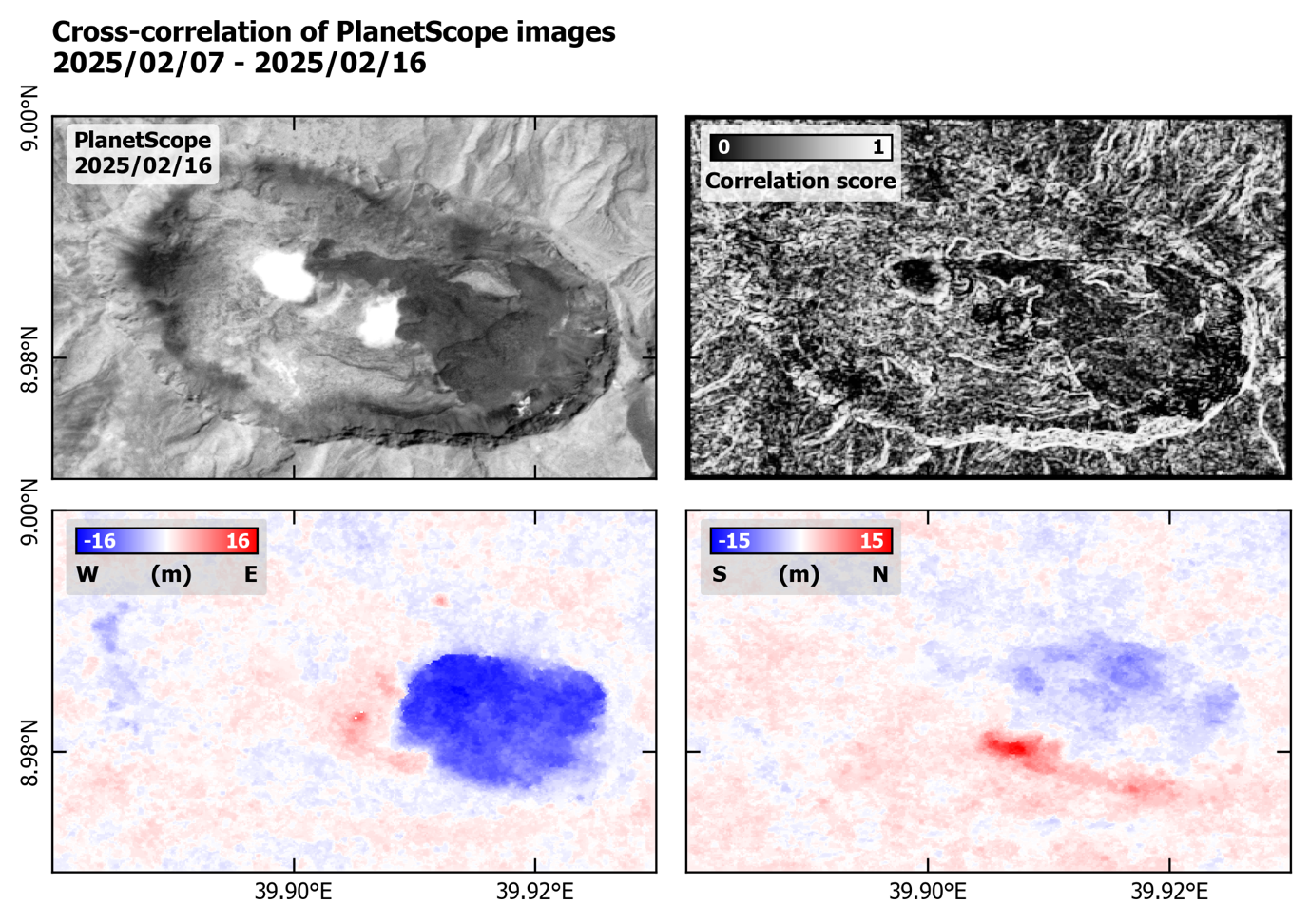


**Fig S7**. (Extended Fig 5 in main text) Horizontal surface displacement from correlation of PlanetScope images acquired on 7^th^ February 2025 and 16^th^ February 2025 (pixel size: 3 m). The upper-left panel shows a panchromatic image acquired on 16^th^ February 2025, showing at least two sites of steam emission. The upper-right panel is the correlation score. In the lower-left panel, negative values (in blue) indicate motion toward the west. In the lower-right panel, negative values (in blue) indicate motion toward the south. The correlation was done using the MicMac software (Rosu et al., 2015). Imagery © 2025 Planet Labs Inc. Modified from COMET Event Response Report 1.7 (18 Feb 2025).


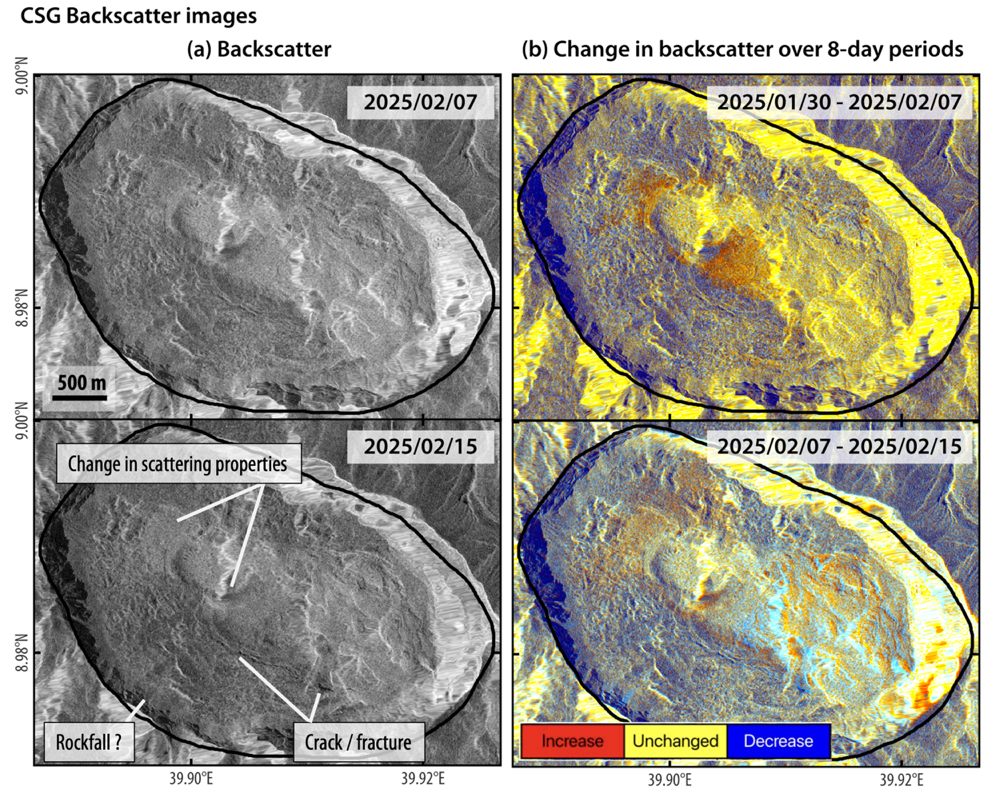


**Fig S8**. (Extended Fig 5 in main text) CSG backscatter data to show changes in ground scattering properties. (a) Comparing individual backscatter images on 7^th^ and 15^th^ February reveals changes that might be related to possible rockfall and formation of cracks. (b) RGB images of change in backscatter over consecutive 8-day periods. Originally published in COMET Event Response Report 1.7 (18 Feb 2025).
